# 527 Opportunity Amongst the Opportunistic: Improving Administration of Time-Critical Medications within a Burn Center

**DOI:** 10.1093/jbcr/irad045.124

**Published:** 2023-05-15

**Authors:** Stacey Richerbach, Tiffany Hockenberry, Karen Richey, Kevin Foster

**Affiliations:** Arizona Burn Center at Valleywise Health, Phoenix, Arizona; AZ Burn Center at Valleywise Health, Phoenix, Arizona; Arizona Burn Center Valleywise Health, Phoenix, Arizona; AZ Burn Center at Valleywise Health, Phoenix, Arizona; Arizona Burn Center at Valleywise Health, Phoenix, Arizona; AZ Burn Center at Valleywise Health, Phoenix, Arizona; Arizona Burn Center Valleywise Health, Phoenix, Arizona; AZ Burn Center at Valleywise Health, Phoenix, Arizona; Arizona Burn Center at Valleywise Health, Phoenix, Arizona; AZ Burn Center at Valleywise Health, Phoenix, Arizona; Arizona Burn Center Valleywise Health, Phoenix, Arizona; AZ Burn Center at Valleywise Health, Phoenix, Arizona; Arizona Burn Center at Valleywise Health, Phoenix, Arizona; AZ Burn Center at Valleywise Health, Phoenix, Arizona; Arizona Burn Center Valleywise Health, Phoenix, Arizona; AZ Burn Center at Valleywise Health, Phoenix, Arizona

## Abstract

**Introduction:**

The absence of skin places patients with burns at an increased risk for a compromised immune system, thus susceptible to opportunistic infection. The use of time-critical medications (TCM) is often required for treatment. By definition, untimely administration may result in suboptimal therapy and put the patient at risk for harm. Our institutional goal for administration is 90%. In 2021 we observed a drop in compliance to 86.8% and instituted a multidisciplinary, quality improvement (QI) approach to improve TCM administration. The purpose of this study was to evaluate the efficacy of our interventions and identify trends in medication administration by staff type.

**Methods:**

QI interventions included a collaboration between Nursing, Pharmacy, and Informatics to overcome barriers to timely administration related to unavailability of medication, education, and options for documentation in the electronic health record (EHR). Medication dispensing systems were floor stocked with eligible TCMs and pharmacists were instructed to refrain from scheduling TCMs within 30 minutes of shift change. Education was disseminated via weekly newsletter, staff huddle, and individual feedback was provided for low performers. TCM metrics were added to the quality board and top performers were acknowledged in a ‘Benchmark Brief.’ Lastly, the EHR was updated to enhance documentation. Medication administration was grouped by year. Staff was identified as burn staff or non-burn staff; comprised of internal resource, contract, and float staff.

**Results:**

Following initiation of the QI project, timely administration increased by 8.6% to a 95.4% rate of timely administration.

**Conclusions:**

Despite increases in annual inpatient admissions, our multidisciplinary quality improvement project was successful, increasing administration of TCM by 8.6%. Further, improvement by burn staff exceeded that of non-burn staff in the burn center. This supports a need to enhance quality initiatives for non-burn staff in the center.

**Applicability of Research to Practice:**

Further research is needed to evaluate efficacy of education, specifically related to temporal dissemination amongst non-burn staff.

